# Urinary Fetuin-A with Specific Post-Translational Modification in Type 1 Diabetes Patients with Normoalbuminuria and Preserved Kidney Function

**DOI:** 10.3390/diagnostics15040423

**Published:** 2025-02-10

**Authors:** Sandra Božičević, Tomislav Bulum, Lea Smirčić Duvnjak, Marijana Vučić Lovrenčić

**Affiliations:** 1 Department of Medical Biochemistry and Laboratory Medicine, Merkur University Hospital, Zajčeva 19, 10000 Zagreb, Croatia; sandra.bozicevic@kb-merkur.hr; 2 Vuk Vrhovac Univerity Clinic, Merkur University Hospital, Zajčeva 19, 10000 Zagreb, Croatia; tomobulum@gmail.com (T.B.); lea.smircic.duvnjak@gmail.com (L.S.D.); 3 Medical Faculty, University of Zagreb, Šalata 2, 10000 Zagreb, Croatia

**Keywords:** type 1 diabetes, diabetic kidney disease, post-translationally modified fetuin-A, biomarker

## Abstract

**Background/Objectives**: Post-translationally modified peptide fragments of fetuin-A (FetA) were identified as a potential biomarker of diabetic kidney disease (DKD). An independent association between urinary FetA-derived peptide levels (uPTM3-FetA) and DKD progression in patients with type 2 diabetes (T2D) was evidenced. This study aimed to explore uPTM3-FetA excretion and its associations with insulin resistance, inflammatory and metabolic biomarkers in patients with type 1 diabetes (T1D), and the normal albuminuria and estimated glomerular filtration rate (eGFR) > 60 mL/min/1.73 m^2^. **Methods**: uPTM3-FetA levels in aliquots of 24 h urine specimens, routine laboratory renal, metabolic and inflammatory tests, adipokines (leptin, adiponectin, resistin), and insulin resistance, assessed as the estimated glucose disposal rate (eGDR), were measured in a cohort of 169 adult T1D patients. To evaluate the changes in early renal dysfunction, the cohort was divided according to the median eGFR. Above- and below-median-eGFR groups were considered as having normal and declining kidney function, respectively. **Results**: The median (IQR) uPTM3-FetA level was 11.7 (8.43–16.65 µg/24 h), with no significant difference between males and females, as well as normal and declining kidney function patients. However, a sex-specific analysis revealed a significantly higher uPTM3-FetA excretion in male T1D patients with lower eGFRs, when compared to those with higher eGFRs, whereas no such difference was observed in female patients. BMI, hs-CRP, resistin and HDL-cholesterol were identified as independent predictors of uPTM3-FetA excretion. **Conclusions**: Our results implicate the potential role of uPTM3-FetA in the detection of an early renal dysfunction in male patients with T1DM and pinpoint the importance of a sex-specific approach in diabetes diagnostics and research.

## 1. Introduction

Annual screening for diabetic kidney disease (DKD) by testing the urinary albumin excretion rate (AER) and serum creatinine-based estimated glomerular filtration rate (eGFR) is one of the cornerstones of diabetes care [1]. DKD is a prevalent complication of diabetes caused by hyperglycaemia-induced glomerular and renal vascular damage [2]. The early detection of renal decline in diabetes is of utmost importance since targeted interventions can efficiently prevent or delay the progression of kidney failure and improve overall patient outcomes [3]. Despite advances in the available interventions, due to the dramatic increase in diabetes incidence, DKD is still the most common cause of end-stage renal disease (ESRD) worldwide and is associated with high cardiovascular morbidity and mortality [4].

DKD is a slow-developing complication of diabetes, affecting patients with both type 1 (T1D) and type 2 diabetes (T2D). The estimated risk for DKD development is 50% for T1D, and 20–50% for T2D, respectively [5]. While DKD develops more than 10 years after diagnosis of T1D, kidney damage may already be present at the time of diagnosis of T2D. This difference is the consequence of a large proportion of undiagnosed T2D older age patients, with a physiological decline in renal function and common comorbidities that also contribute to kidney damage, such as obesity and hypertension [6].

The traditional view on DKD pathogenesis implicates hyperfiltration and albuminuria as the first sign of early glomerular damage, followed by overt proteinuria and a decline in GFR along with progressive renal decline [2]. However, this concept has been challenged by accumulating evidence on a substantial proportion of patients with a significant decline in eGFR in the absence of an elevated AER [7]. This non-albuminuric DKD phenotype seems to be associated with tubular, rather than glomerular damage, and a higher cardiovascular risk [8]. Furthermore, there are subsets of patients with DKD who rapidly progress to ESRD, but also those who successfully restore their kidney function due to a favourable response to treatment [9]. Although some clinical risk factors such as age, diabetes duration, blood pressure and level of hyperglycaemia have been associated with various phenotypes of DKD, the addition of clinical factors to albuminuria and eGFR only modestly improved the prediction of GFR decline [10,11]. Therefore, additional biomarkers are needed for the early detection of and differentiation between various phenotypes of DKD, enabling targeted interventions to maintain kidney function and reduce risks of adverse outcomes.

Large-scale urinary proteomic studies have identified post-translationally modified peptide fragments derived from fetuin-A (uPTM3-FetA) as a potential biomarker of chronic kidney disease (CKD) [12,13]. Fetuin-A is a hepatokine with a physiological role in the regulation of mineral metabolism and prevention of ectopic calcification [14], but it is also implicated as a risk factor of diabetes, insulin resistance, inflammation and fibrosis, all of which contribute to kidney damage [15]. Urinary fetuin-A is associated with chronic histological damage and adverse clinical outcomes across a variety of biopsy-proven kidney diseases [16], and an increased excretion of uPTM3-FetA in patients with CKD, regardless of the diabetes diagnosis, was found [17]. A recent prospective study by Chuang et al. demonstrated the independent association of uPTM-FetA with the development of DKD and its significant predictive power over traditional biomarkers, albuminuria and eGFR, in detecting kidney function deterioration in two independent cohorts of T2D patients [18]. The possible role of uPTM3-FetA in the early detection of renal decline in T1D has not been studied so far.

This study aimed to explore the associations of uPTM3-FetA with inflammatory, metabolic and renal variables, as well as insulin resistance in T1D patients with normal albuminuria and glomerular filtration rate. We hypothesised that uPTM-FetA excretion may reflect an early decline in kidney function in T1D patients at the clinical stage which is usually considered normal by traditional diagnostic tools.

## 2. Materials and Methods

### 2.1. Patients

We conducted this cross-sectional study at a tertiary diabetes care centre and a clinical laboratory affiliated with the Merkur University Hospital in Zagreb, Croatia. The study included adult (18–70 years of age, all Caucasians from Croatia) patients with T1D and normal albuminuria (<30 mg/24 h) and eGFR > 60 mL/min/1.73 m^2^, without or with earlier stages of microvascular complications (non-proliferative diabetic retinopathy, and/or the first degree of peripheral neuropathy, as assessed by a trained ophthalmologist and neurologist) at the time of recruitment. Pregnant and lactating women, as well as patients with established macrovascular complications, malignant diseases, liver dysfunction (assessed by laboratory abnormalities of liver function tests) and/or recent (within 3 months) infectious diseases were not included in the study. T1DM was defined according to islet-cell autoantibody-positivity, a younger age (below 35 years) at diagnosis, and insulin treatment initiated within one year of diagnosis.

The patients were recruited at their annual follow-up consultations after giving informed consent. The information on demographics, medical history and treatment, as well as routine laboratory workups, were obtained from electronic patient records. Blood samples were collected from fasting patients during their annual follow-up visits. The patients collected 24 h urine samples for quantitative albuminuria and proteinuria, according to written instructions provided by the Croatian Society of Medical Biochemistry and Laboratory Medicine available at https://www.hdmblm.hr/hr/informacije-za-pacijente/pitanja, accessed on 1 February 2025) as a routine part of their annual follow-up. The complete amount of urine excreted within 24 h was delivered to the laboratory, where the volume was measured with a graduated cylinder. Routine laboratory tests were performed immediately, while aliquots of serum for adiponectin, leptin, resistin, and 24 h urine collection for uPTM-FetA were stored at −30 °C until they were analysed.

The study was conducted according to the Declaration of Helsinki and approved by the Ethics Committee of the Merkur University Hospital (0311-2172-13/February 2019).

### 2.2. Laboratory Testing

Routine biochemistry, haematology, and HbA1c analyses were carried out in a clinical laboratory with accredited procedures [19] on dedicated platforms: AU680 (Beckman Coulter, Brea, CA, USA), XN1000 (Sysmex Corp., Kobe, Japan) and Integra 400 (Roche Diagnostics, Zug, Switzerland).

Serum adiponectin, leptin and resistin were tested with dedicated ELISA-assays (Adiponectin Human ELISA-High Sensitivity, Leptin Human ELISA-Clinical Range, and Resistin Human ELISA, respectively; all from BioVendor, Brno, Czech Republic) following the manufacturer’s instructions.

### 2.3. Estimates of Glomerular Filtration Rate and Insulin Resistance

Glomerular filtration rate (eGFR) was estimated by a 2009-creatinine-based four-parameter CKD-EPI equation [20]. Insulin sensitivity was assessed as an estimated glucose disposal rate (eGDR) by using a validated formula based on clinical variables (waist circumference and hypertension) and HbA1c. The degree of insulin resistance is inversely related to the size of the eGDR [21].

### 2.4. Measurement of Post-Translationally Modified Fetuin-A Fragments in Urine

uPTM-FetA concentration was measured in aliquots of 24 h urine samples routinely collected at the patient’s annual checkup, using IVD/CE certified uPTM3-FetA ELISA kits (DNlite-IVD103-Human uPTM3-FetA-DKD ELISA, Bio Preventive Med Corp., Zubei City, Taiwan) according to the manufacturer’s instructions. Our previous pilot study revealed the total imprecision of the assay (expressed as coefficient of variation—CV) of 12.3% and 7.6% at the uPTM3-FetA levels of 4.5 and 12.3 μg/L, respectively. The results are expressed as the daily excretion rate of uPTM3-FetA (μg/24 h). [22]

### 2.5. Data Analysis and Statistics

The Shapiro–Wilk test was used to test data distribution normality, and outliers were tested by the IQR method. The results of continuous variables are presented as means (standard deviation), or medians (IQR), depending on the normality of distribution, whereas categorical variables are expressed as frequency/percentages. Appropriate parametric (two-tailed Student’s *t* test) and non-parametric tests (Mann–Whitney U test) were performed to evaluate the differences in continuous variables, and a Chi-squared test was used for the testing of categorical variable differences between the groups. Spearman’s rank correlation was used to determine possible associations between different parameters, followed by a backward multiple linear regression analysis to evaluate the determinants of uPTM-FetA levels in patients with T1D. A logistic regression analysis was used to determine independent risk factors for the early eGFR decline. The level of statistical significance was defined at *p* < 0.05. The statistical analysis was carried out by the MedCalc^®^ Statistical Software version 19.6 (MedCalc Software Ltd., Ostend, Belgium; https://www.medcalc.org; 2020).

## 3. Results

### 3.1. Study Population

A total of 169 T1D patients (M/F = 96/73), aged 18–79 years, with diabetes for a duration ranging from 1 to 47 years, with complete clinical and laboratory evaluations were included in the study. The original sample size was 180; however, we excluded cases with incomplete data. The median (IQR) level of uPTM3-FetA was 11.7 (8.43–16.65 µg/24 h), with no significant difference between female [11.48 (8.10–16.45) µg/24 h] and male [12.15 (8.95–17.49) µg/24 h] patients (*p* = 0.4056; Supplementary Table S1).

### 3.2. Relationship Between uPTM3-FetA and Glomerular Filtration Rate in Patients with T1D

To evaluate the influence of an early renal dysfunction in the study cohort of patients with T1D, normal albuminuria (<30 mg/day) and eGFR (>60 mL/min/1.73 m^2^), we divided patients according to the median eGFR (97 mL/min/1.73 m^2^ for females, and 103 mL/min/1.73 m^2^ for males). An eGFR above the median was considered normal, whereas an eGFR below the median indicated a decline in kidney function. The patients with higher eGFRs (48% females and 55% males) were significantly younger and had a shorter duration of T1D but had unfavourable profiles of inflammatory and metabolic biomarkers: higher WBC and HbA1c, and lower HDL-C and adiponectin levels, respectively. Urinary albumin, BMI, insulin resistance (assessed as eGDR), CRP, LDL-cholesterol, triglycerides, leptin and resistin did not differ between the median-eGFR categories (Table 1).

**Figure 1:**
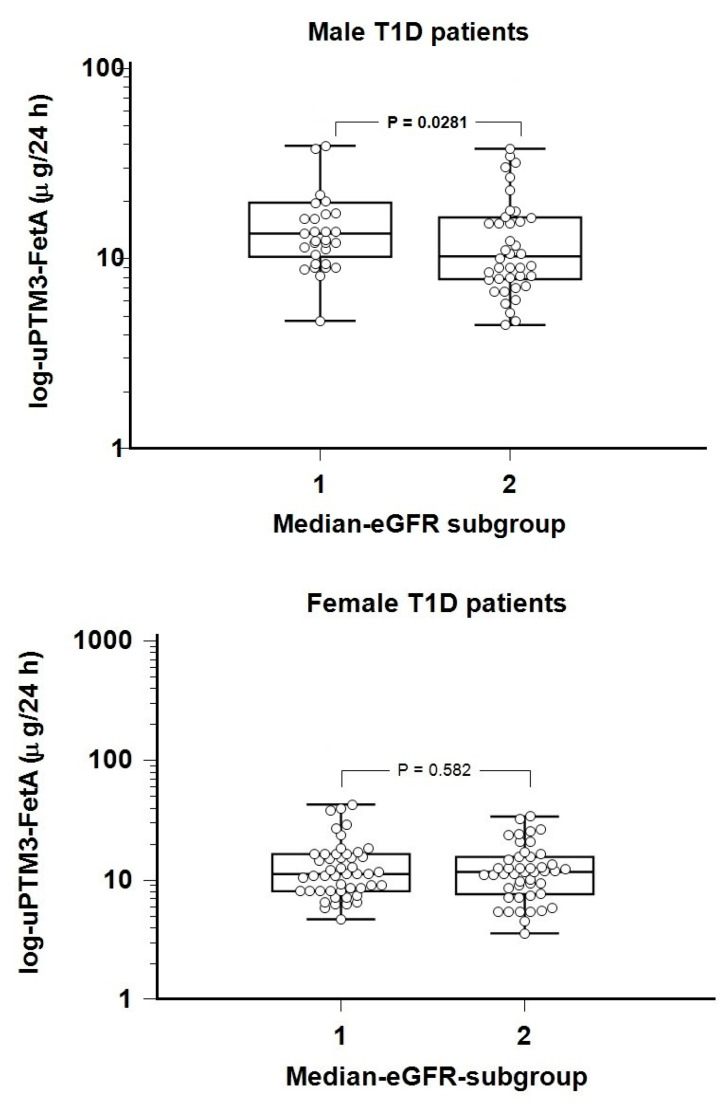
Log-transformed uPTM3-FetA levels in subgroups of normoalbuminuric patients with type 1 diabetes, and lower (1) and higher (2) eGFR, respectively, according to sex. Mann–Whitney U test data are expressed as median (IQR). eGFR—estimated glomerular filtration rate, uPTM3-FetA—urinary post-translationally modified fetuin-A fragments.

Also, no statistically significant difference in uPTM3-FetA levels was observed between patients sub-grouped according to the median eGFR (0.069; Supplementary Table S1). However, a sex-specific analysis revealed a significantly higher uPTM3-FetA excretion in male patients with a lower eGFR, when compared to those with a normal eGFR, whereas no such difference was evidenced in the two subgroups of female patients (Figure 1).

Considering the observed sex-related difference, an additional analysis of data was carried out according to biological sex. There were no significant differences in uPTM3-FetA excretion, as well as diabetes duration, albuminuria, HbA1c, CRP, WBC, LDL-c, triglycerides and resistin levels between female and male patients. However, the female patients were significantly older, had a lower BMI and eGFR, and higher insulin sensitivity, adiponectin, leptin and HDL-c levels than male patients with T1D. The data are presented in Supplementary Table S1.

### 3.3. Correlation of uPTM-FetA Excretion with Kidney Function, Glycaemia, and Insulin Sensitivity

**Figure 2:**
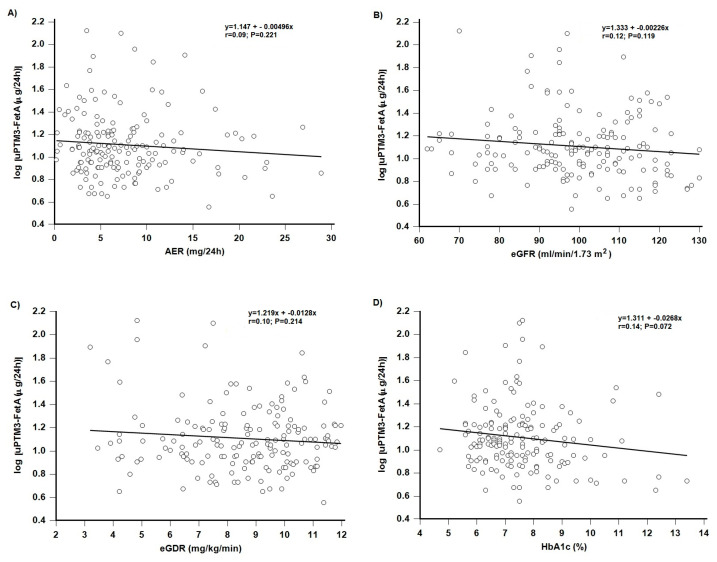
Scatterplots of log-transformed uPTM3-FetA levels in normoalbuminuric patients type 1 diabetes and albuminuria (**A**), eGFR (**B**), eGDR (**C**) and HbA1c (**D**), obtained by linear regression analysis. AER: albumin excretion rate, eGFR: estimated glomerular filtration rate, eGDR: estimated glucose disposal rate, uPTM3-FetA: urinary post-translationally modified fetuin-A fragments.

The relationship between u-PTM3-FetA excretion and the variables of kidney function, albuminuria and eGFR, average glycaemia and insulin sensitivity (HbA1c and eGDR), was tested by a simple linear regression analysis. No significant relationships were observed by any of the tested variables in patients with T1D, normoalbuminuria, and preserved kidney function (Figure 2).

### 3.4. Determinants of uPTM-FetA Excretion in Patients with T1D

A backward multiple linear regression analysis was performed to examine the determinants of uPTM3-FetA excretion. The model included logarithmically transformed uPTM3-FetA values, and age, diabetes duration, BMI, HbA1c, HDL- and LDL-cholesterol, triglycerides, adiponectin, leptin, resistin, CRP, WBC, AER, eGFR, and liver function tests (AST, ALT, GGT, alkaline phosphatase, and bilirubin), respectively, as independent variables, and biological sex as a covariate. The model identified BMI, CRP, HDL, and resistin levels as significant factors, whereas the other variables did not influence uPTM3-FetA excretion. The model was statistically significant (F = 3.8785, *p* = 0.0024); however, an R^2^ value of 0.108 indicates that it was able to explain only 11% of the uPTM3-FetA variance in the study cohort of T1D patients (R^2^ = 0.108). The results are presented in Table 2.

### 3.5. Predicting Variables of eGFR Decline in Patients with T1D and Normoalbuminuria

A logistic regression analysis was carried out to assess the predicting variables of eGFR decline in T1D patients with normoalbuminuria and eGFR > 60 mL/min/1.73 m^2^. The model included below- and above-median-eGFR categories (97 mL/min/1.73 m^2^ for females, and 103 mL/min/1.73 m^2^ for males) as a binary dependent variable, and log-transformed uPTM3-FetA, adiponectin, leptin, resistin, HDL-cholesterol, hs-CRP, WBC, AER, eGDR, HbA1c and diabetes duration, respectively, as continuous independent variables (Table 2), with sex as a biological confounder. The model was statistically significant (χ^2^ = 36.39; *p* < 0.001) and was able to explain 26.1% of the variance (Nagelkerke R^2^).

AER and HDL-cholesterol were significant positive factors, while eGDR and HbA1c were negative factors independently associated with eGFR decline. The model correctly classified 67% of the cases and provided the area under the ROC curve as 0.754 (CI: 0.682 to 0.817).

Given the observed difference in uPTM3-FetA levels regarding eGFR levels between male and female patients, we also conducted separate logistic regression analyses according to sex. A significant effect of HbA1c regarding eGFR decline was observed in both male and female patients, while the effects of albuminuria, eGDR, and HDL-cholesterol remained significant in women, but not in men. On the other hand, the model retained log-uPTM3-FetA levels and eGDR as factors affecting renal decline in male patients T1D, albeit with marginal significance (Table 3).

## 4. Discussion

In this study, we investigated an emerging biomarker of DKD, uPTM3-FetA, in relation to early renal decline in patients with T1D and normal kidney function as assessed by traditional diagnostic tools: albuminuria and eGFR. The excretion of uPTM3-FetA was significantly higher in male patients with lower eGFRs. In contrast, no differences in uPTM3-FetA excretion according to eGFR levels were observed in female patients with T1D. Albuminuria, HDL-cholesterol, IR-eGDR, and HbA1c were identified as significant predictors of renal decline in female patients. In contrast, renal decline in male patients with T1D could be predicted by HbA1c only, with marginally significant effects of eGDR and uPTM3-FetA excretion. To our knowledge, this is the first study addressing the possible role of uPTM3-FetA in detecting early changes in kidney function in patients with T1D.

Urinary FetA emerged as a biomarker of DKD in proteomic studies, but the excretion of specific post-translationally modified fragments (uPTM3-FetA) has only been studied in patients with T2D so far [13,17,18]. FetA came into the focus of diabetes research since it enhances common risk factors of T2D such as insulin resistance, liver steatosis and inflammation. However, despite multiple observational studies which implicated a strong positive association between circulating FetA levels and the risk of T2D and its complications [23,24], a causal relationship was not confirmed [25]. Interestingly, urinary FetA excretion is unrelated to the circulating FetA levels. It was proposed that FetA is excreted in urine due to glomerular injury, but tubular synthesis was also evidenced upon kidney damage as a hypothesised protective mechanism [18]. In healthy adults, fetuin-A is not expressed in the kidneys, ref. [26] indicating that measurable levels of uPTM3-FetA are absent in this population. Consequently, a direct comparison of the results between healthy individuals and those with diabetes seem to be not feasible for uPTM3-FetA.

The independence of urinary excretion from circulating FetA levels indicates a high kidney-specificity of excreted biomarkers, regardless of the diabetes type. Our study was not designed to explore the circulating FetA levels, but a lack of the effects of routine variables of hepatic function indicates that uPTM3-FetA excretion is rather an indicator of kidney-specific changes, than a reflection of hepatic FetA synthesis. Our results reveal a weak, but significant positive association of uPTM3-FetA excretion with BMI, HDL-cholesterol, and resistin levels, and a negative association with circulating hs-CRP levels. While these results support the hypothesis that uPTM-FetA excretion is partly related to obesity and metabolic syndrome variables [15], a lack of association with eGDR, adiponectin, and leptin, and the weak effect size of the linear regression model indicate that, in this cross-sectional study, other unidentified factors determine uPTM3-FetA excretion in patients with T1D, normoalbuminuria, and preserved kidney function.

However, the aim of this study was to explore uPTM3-FetA excretion in the context of early renal decline at a clinical stage which is usually considered as normal according to the traditional tools for diagnosis and classification of DKD. DKD in T1D is associated with an older age and a more advanced course of the disease. This is confirmed in our study as well, since patients with lower eGFRs were older, and had a longer duration of diabetes in comparison to those with higher eGFRs. Also, the use of antihypertensive medication and statins was significantly higher in this subgroup. Higher HDL-cholesterol and adiponectin levels in our renal declining group are consistent with the evidence of an independent association of the higher HDL-C levels with accelerated GFR loss, and a relationship between elevated adiponectin levels and renal dysfunction [27,28]. However, our finding on a lower WBC count in a group of T1D patients with lower eGFRs is not consistent with the evidence on the activation of immune and inflammatory pathways in the development and progression of DKD [29]. Given the fact that T1D is associated with a lower WBC count [30], and the methodological limitation of our study used a composite leukocyte count instead of WBC subpopulation analysis, this observation warrants further investigation. Our findings on higher HbA1c levels in the group of T1D patients with higher eGFRs are not in line with the evidence implicating HbA1c levels as a strong, concentration-dependent predictor for eGFR decline [31,32]. It is possible that the observed discrepancy arises from the design of our study, which included only T1D patients with normoalbuminuria and preserved kidney function and who were free from other advanced microvascular complications. Given the median duration of diabetes (14 years) in our cohort, this could have been attained only by the rigorous control of glycaemia, as reflected by the near-target median HbA1c (7.3%). Nevertheless, T1D patients from both subgroups would benefit from attaining and maintaining target HbA1c levels (<7.0%) and avoiding glycaemic variability, another established risk factor for DKD progression in T1D [33].

The logistic regression analysis also revealed an interesting sex-dimorphic pattern of renal decline in patients with T1D and normoalbuminuria. Lower eGFR levels were associated with albuminuria, HDL-cholesterol and eGDR in female but not in male patients with normal kidney function. HbA1c was a significant negative predictor in both sexes, but this finding should be taken into account with the limitations related to the study design discussed above. Although eGDR and uPTM3-FetA were retained in the predictive model of renal decline in male patients, the effects failed to attain statistical significance, which may be due to a relatively smaller number of male patients in our study cohort. Thus, additional research with larger sample sizes should be carried out to confirm the independent effects of uPTM3-FetA in early renal decline in male T1D patients with normoalbuminuria.

Apart from the sample size, our study is limited by a singular ethnicity, as well as a relatively wide age range and the diabetes duration among the included patients. Also, a cross-sectional study design has limitations as it does not provide insight into the causal relationships between variables and the changes over time. Finally, our study did not include patients with DKD to evaluate uPTM3-FetA excretion in patients with T1D across the various stages of kidney disease.

Nevertheless, our data suggest that uPTM3-FetA may reflect the early events in DKD development in patients with T1D and normoalbuminuria in a sex-dimorphic pattern.

Gender and biological sex differences in the epidemiology and pathogenesis of diabetes and its complications elicit a significant scientific interest in the concept of personalised medicine. Epidemiological studies indicate that T1D is not only more prevalent in males than females [34], but the male sex appears to be associated with a higher risk of DKD and its progression to ESRD [35]. An increasing number of epidemiological and clinical studies suggest that sex hormones play a significant role in the onset and progression of DKD [36]. The protective effects of oestrogens, mainly through oestrogen receptor α activation in various tissues, including kidney and pancreatic beta cells, as well as the effects of sex chromosomes, fetal/neonatal programming, and epigenetic modifications are all implicated in the sex-related variability in diabetes [37]. On the other hand, testosterone has the potential to exacerbate DKD by activating the renin–angiotensin–aldosterone system (RAAS) which can lead to the induction of tubular fibrosis [38]. This mechanism might contribute to the tubular expression of fetuin-A and, consequently, to the increased excretion of uPTM-FetA in male patients observed in our study. However, this remains unclear, as our study did not include the determination of circulating sex hormones.

While the precise molecular mechanisms by which sex hormones affect the pathophysiology of DKD remain largely unclear, our findings suggest a potential role for uPTM3-FetA in the early detection of renal dysfunction in male patients with T1DM, as well as highlight the relevance of considering a biological sex-specific approach in DKD diagnostics and research. Further prospective studies on uPTM3-FetA excretion, including its relationship with reproductive hormones across various patient populations, are warranted to clarify the relevance of uPTM3-FetA in the pathogenesis and early detection of kidney disease in patients with type 1 diabetes.

## Figures and Tables

**Table 1:** Clinical characteristics of the study cohort of normoalbuminuric patients with type 1 diabetes, classified by estimated glomerular filtration rate.

	Total	eGFR Below Median	eGFR Above Median	*p*
Age (years)	45 (18–70)	53 (23–70)	36 (18–66)	0.001
Diabetes duration (years)	14 (1–47)	18 (2–47)	12 (1–41)	<0.001
Female/Male [N(%)/N(%)]	96 (57)/73 (43)	50 (60)/33 (40)	46 (53)/40 (47)	0.377
BMI (kg/m^2^)	24.7 (22.2–27.2)	25.1 (22.5–27.3)	24.6 (21.6–27.1)	0.110
HbA1c (%)	7.3 (6.6–8.1)	7.2 (6.4–7.7)	7.5 (6.6–8.8)	0.018
HDL-cholesterol (mmol/L)	1.70 ± 0.37	1.80 ± 0.40	1.60 ± 0.32	0.001
LDL-cholesterol (mmol/L)	2.98 (2.55–3.36)	2.87 (2.45–3.32)	3.07 (2.68–3.50)	0.097
Triglycerides (mmol/L)	0.86 (0.70–1.18)	0.84 (0.70–1.12)	0.54 (0.70–1.21)	0.570
AER (mg/24 h)	6.20 (3.88–9.77)	6.1 (3.9–10.5)	6.2 (3.8–9.1)	0.522
eGFR (mL/min/1.73 m^2^)	99 (91–111)	90 (79–95)	111 (105–117)	<0.001
hs-CRP (mg/L)	1.10 (0.60–2.48)	1.20 (0.63–2.48)	1.10 (0.55–2.45)	0.604
WBCs (10^9^/L)	6.2 (5.3–7.4)	5.9 (5.12–6.9)	6.5 (5.6–8.0)	0.009
eGDR (mg/kg/min)	8.87 (7.29–10.10)	8.66 (6.43–10.08)	9.05 (7.77–10.22)	0.067
Adiponectin (µg/L)	11.05 (7.07–15.84)	12.91 (8.29–17.93)	9.77 (6.47–14.03)	0.010
Leptin (ng/L)	8.26 (4.11–18.06)	9.46 (4.38–18.41)	7.18 (3.84–17.00)	0.208
Resistin (µg /L)	4.86 (3.90–6.34)	4.83 (4.07–6.16)	4.89 (3.77–6.52)	0.810
uPTM3-FetA (µg/24 h)	11.7 (8.43–16.65)	12.2 (9.0–17.1)	11.17 (7.80–15.75)	0.069
Antihypertensive therapy				
ACEi/ARB [N(%)]	73 (43)	46 (55)	25 (29)	<0.001
CCB [N(%)]	17 (10)	(14) 17	2 (2)	<0.001
Statins [N(%)]	66 (39)	44 (53)	20 (23)	<0.001

The data are presented as median [range], median (IQR) and mean ± SD, as appropriate. BMI—body mass index, AER—albumin excretion rate, eGFR—estimated glomerular filtration rate, hs-CRP—high-sensitivity c-reactive protein, WBCs—white blood cells, eGDR—estimated glucose disposal rate, uPTM3-FetA—urinary post-translationally modified fetuin-A fragments, ACEi/ARB—angiotensin converting enzyme inhibitors/angiotensin receptor blockers, CCB—calcium channel blockers.

**Table 2:** Determinants of log-transformed uPTM3-FetA levels in normoalbuminuric type 1 diabetes patients.

Variable	β	*p*
BMI (kg/m^2^)	0.015	0.021
hs-CRP (mg/L)	−0.019	0.022
HDL-cholesterol (mmol/L)	0.144	0.014
Resistin (µg /L)	0.024	0.049

Backward multiple regression analysis. BMI—body mass index, hs-CRP—high-sensitivity c-reactive protein; β—coefficient of the relationship between each predictor variable and the log-odds of the outcome. Variables included in the model: age, diabetes duration, BMI, HbA1c, HDL- and LDL-cholesterol, triglycerides, adiponectin, leptin, resistin, CRP, WBC, AER, eGFR, and liver function tests (AST, ALT, GGT, alkaline phosphatase, and bilirubin), with biological sex as a covariate.

**Table 3:** Multivariate logistic analysis of the factors associated with eGFR decline in normoalbuminuric patients with type 1 diabetes.

	Total		Females		Males	
Variable	Odds Ratio (95% CI)	*p*	Odds Ratio (95% CI)	*p*	Odds Ratio (95% CI)	*p*
AER (mg/24 h)	1.085 (1.014–1.160)	0.018	1.161 (1.046–1.288)	0.005	Not retained in the model	
HDL-cholesterol (mmol/L)	6.637 (2.278–19.339)	<0.001	6.758 (1.240–36.842)	0.027	Not retained in the model	
HbA1c (%)	0.611 (0.458–0.815)	<0.001	0.458 (0.287–0.732)	0.001	0.649 (0.433–0.971)	0.035
eGDR (mg/kg/min)	0.725 (0.605–0.868)	<0.001	0.645 (0.484–0.861)	0.003	0.7871 (0.602–1.029)	0.081
uPTM3-FetA (µg/24 h)	Not retained in the model		Not retained in the model		5.3487 (0.786–36.393)	0.087

AER—albumin excretion rate, eGDR—estimated glucose disposal rate, uPTM3-FetA—urinary post-translationally modified fetuin-A fragments.

## Data Availability

The data underlying this article are available in the article and can be obtained from the authors upon reasonable request.

## Supplementary Materials

The following supporting information can be downloaded at: https://www.mdpi.com/article/10.3390/diagnostics15040423/s1, Table S1. Clinical characteristics of the study cohort of normoalbuminuric type 1 diabetes patients with normal kidney function, classified by biological sex.

## Abbreviations

The following abbreviations are used in this manuscript:

T1D	Type 1 diabetes
T2D	Type 2 diabetes
DKD	Diabetic kidney disease
CKD	Chronic kidney disease
AER	Albumin excretion rate
eGFR	Estimated glomerular filtration rate
uPTM3-FetA	Urinary post-translationally modified fetuin-A fragments
eGDR	Estimated glucose disposal rate
hs-CRP	High-sensitivity c-reactive protein
WBC	White blood cells
