# Supplementary material for: Urinary Fetuin-A with Specific Post-Translational Modification in Type 1 Diabetes Patients with Normoalbuminuria and Preserved Kidney Function
Source: Diagnostics (Basel). 2025 Feb 10;15(4):423. doi: 10.3390/diagnostics15040423 (PMC11854771; doi:10.3390/diagnostics15040423)
Supplement: Supplementary file 1 [file diagnostics-15-00423-s001.zip › diagnostics-3432022-supplementary.pdf]

Supplementary Table S1. Clinical characteristics of the study cohort of normoalbuminuric type 1 diabetes patients with normal kidney function, classified by biological sex.

	Females (N=96)	Males N=73)	P
**Age (years)**	**49 [18 - 70]**	**37 [18 - 67]**	**<0,001**
Diabetes duration (years)	14 [1 – 47]	13 [1 – 38]	0,480
**BMI (kg/m²)**	**24,1 (21,7 – 25,8)**	**25,4 (23,2 – 27,8)**	**0,007**
HbA1c (%)	7,6 (6,6 – 8,3)	7,1 (6,5 – 7,8)	0,079
**HDL-cholesterol (mmol/L)**	**1,84 ± 0,35**	**1,52 ± 0,32**	**<0,001**
LDL-cholesterol (mmol/L)	2,93 (2,43 – 3,26)	3,07 (2,63 – 3,50)	0,083
Triglycerides (mmol/L)	0,86 (0,68 – 1,12)	0,92 (0,73 – 1,21)	0,145
AER (mg/24h)	5,85 (3,45 – 10,00)	6,50 (4,15 – 9,23)	0,373
**eGFR (mL/min/1.73 m²)**	**90 (84 – 107)**	**103 (94 - 114)**	**0,004**
CRP (mg/L)	1,20 (0,60 – 2,70)	0,95 (0,50 – 2,15)	0,124
WBC (10^9/L)	6,2 (5,1 -7,5)	6,3 (5,4 – 7,3)	0,580
**eGDR (mg/kg/min)**	**9,64 (7,44 – 10,64)**	**8,32 (6,80 – 9,45)**	**0,001**
**Adiponectin (µg/L)**	**14,10 (11,34 – 18,68)**	**7,40 (5,59 – 10.12)**	**<0,001**
**Leptin (ng/L)**	**14,48 (7,93 – 24,0)**	**4,06 (2,25 – 7,51)**	**<0,001**
Resistin (µg /L)	4,86 (4,05 – 6,35)	4,86 (3,88 – 6,34)	0,806
uPTM3-FetA (µg/24h)	11,48 (8,10 – 16,45)	12,15 (8,95 – 17,49)	0,406

Data are presented as median [range], median (IQR) and mean±SD, as appropriate. BMI: body mass index, AER: albumin excretion rate, eGFR: estimated glomerular filtration rate, hs-CRP: high-sensitivity c-reactive protein, WBC: white blood cells, eGDR:estimated glucose disposal rate, uPTM3-FetA: urinary post-translationally modified fetuin A fragments.
